# Association between diabetic control and progression of monoclonal gammopathy of undetermined significance to multiple myeloma

**DOI:** 10.1038/s41408-026-01592-x

**Published:** 2026-08-03

**Authors:** Lawrence Liu, Byron Sigel, Mei Wang, Nikhil Grandhi, Martin W. Schoen, Kristen M. Sanfilippo, Kenneth R. Carson, Murali Janakiram, Tzeyu L. Michaud, Graham A. Colditz, Theodore S. Thomas, Su-Hsin Chang

**Affiliations:** 1 Research Service, St. Louis Veterans Affairs Medical Center, St. Louis, MO USA; 2 Department of Hematology/Oncology, Cedars-Sinai Medical Center, Los Angeles, CA USA; 3 Department of Medicine, Washington University School of Medicine, St. Louis, MO USA; 4 Division of Public Health Sciences, Department of Surgery, Washington University School of Medicine, St. Louis, MO USA; 5 The University of Texas MD Anderson Cancer Center, Houston, TX USA; 6 Division of Hematology and Medical Oncology, Department of Internal Medicine, Saint Louis University School of Medicine, St. Louis, MO USA; 7 Division of Hematology and Oncology, Northwestern University Feinberg School of Medicine, Chicago, IL USA; 8 Department of Hematology & Hematopoietic Cell Transplantation, City of Hope Comprehensive Cancer Center, Duarte, CA USA; 9 Department of Health Promotion, College of Public Health, University of Nebraska Medical Center, Omaha, NE USA

**Keywords:** Risk factors, Myeloma

## Abstract

Diabetes Mellitus (DM) is a risk factor for multiple myeloma (MM), but the relationship between diabetic control and risk of progression to MM in patients with monoclonal gammopathy of undetermined significant (MGUS) is unclear. We conducted a retrospective cohort study of US Veterans diagnosed with MGUS from 10/1/1999-12/31/2023 with prior DM. MGUS and MM diagnoses were confirmed using published natural language processing-assisted models. The exposure was time-varying glycated hemoglobin (HbA1c) > 7% during the follow-up (time of MGUS to MM, death, or being censored on 4/15/2025, whichever occurred first). The outcome was time from MGUS to MM. The association was estimated by multivariable-adjusted hazard ratio (aHR) using a Fine-Gray subdistribution hazard model with death as a competing event, adjusted by inverse probability of treatment weighting (IPTW). Among 11,061 patients with DM, HbA1c > 7% was associated with increased progression risk (aHR 1.15; 95% confidence interval [CI] 1.01–1.32). This association was stronger in the subgroups of Type-2 DM (T2DM) with insulin use (*n* = 7070; aHR 1.46; 95% CI 1.19–1.78) and non-use (*n* = 3724; aHR 1.42; 95% CI 1.14–1.77) throughout follow-up. For patients with T2DM and MGUS, poorly controlled diabetes may be associated with progression risk to MM. Future studies are needed to confirm this relationship.

## Introduction

Multiple myeloma (MM), is a hematologic malignancy leading to an estimated 36,110 new cases and 12,030 deaths in the United States in 2025 [1]. Patients with MM often face significant morbidity and mortality [2], and high cost of care [3].

MM uniformly follows Monoclonal Gammopathy of Undetermined Significance (MGUS), diagnosed or undiagnosed, which affects 3–10% of people aged 50 or older [4]. Individuals with MGUS have a 1% annual risk of progression [5, 6]. MGUS is categorized into risk levels based on: serum monoclonal protein (M-protein) levels of ≥1.5 g/dL, the presence of non-immunoglobulin (Ig) G isotypes, and irregular serum-free light-chain ratios. Furthermore, clinical risk factors include old age, male sex, black race, obesity, and family history [7], among which obesity has been identified as a significant modifiable risk factor [8–11]. Identifying modifiable risk factors is invaluable for future research on preventing or delaying MGUS to MM progression.

Diabetes mellitus (DM) is a common comorbidity present with MGUS [12]. Several studies have shown that DM is a major risk factor for MM disease progression, and worse survival [13–20]. The potential mechanisms include insulin and insulin-like growth factor-1 promoting the survival and proliferation of plasma cells [21–26]. Additionally, previous research suggests that some antidiabetic medications, e.g., metformin and glucagon-like peptide-1 receptor agonists (GLP-1RA), are linked to reduced risk of progression to MM in patients with DM and MGUS, possibly related to diabetic control [14, 15, 27]. Given that insulin resistance and high levels of endogenous insulin are hallmarks of Type 2 DM (T2DM), it raises the question of whether poor diabetic control can increase risk for MGUS progression [28].

Despite the association between DM and MGUS progression, the impact of diabetic control on progression remains uncertain. Additionally, the management of MGUS involves regular monitoring and follow-up, with no guidelines addressing the treatment of DM [29]. Therefore, this study aimed to determine whether diabetic control is associated with the MGUS progression to MM in patients with DM.

## Methods

### Data, study population, and design

**Fig. 1:**
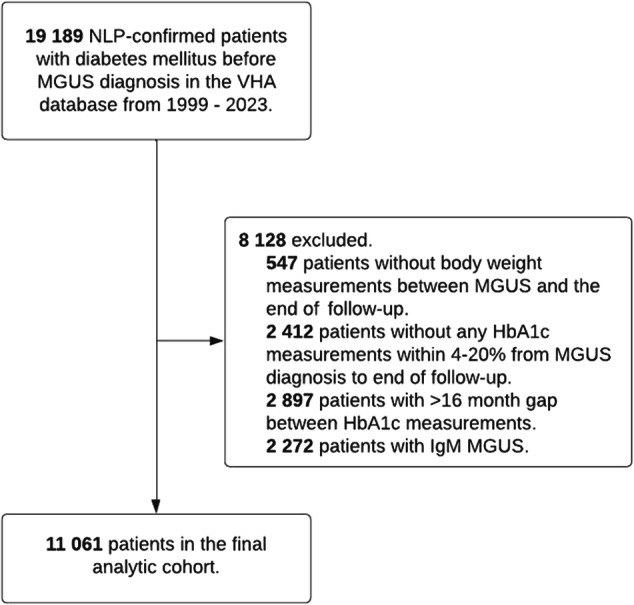
Flowchart of patient accrual for patients diagnosed with MGUS with a prior DM diagnosis in the VHA, 1999–2023.

This population-based cohort study utilized the US Veterans Health Administration (VHA) database, the largest integrated health care system incorporating 170 medical centers across the nation. Patients diagnosed with MGUS from 10/1/1999-12/31/2023 with a prior diagnosis of Type 1 DM (T1DM) or T2DM were included (Fig. 1). T1DM was included to allow for comprehensively assessing any potential relationship between diabetic control and MGUS progression, as prior work demonstrated increased cancer risk with higher insulin dose levels [30].

MGUS and MM diagnoses were confirmed via a published natural language-processing (NLP)-assisted machine learning algorithm [31]. DM diagnosis was identified via International Classification of Diseases (ICD)-9/10 codes and DM treatment (Supplementary Table 1) [31].

We excluded patients: (1) without body weight measurements between MGUS and the end of follow-up to exclude patients who sought care outside of the VHA; (2) with HbA1c value < 4% or >20% during the follow-up for potential documentation errors in their laboratory results; (3) with >16 month (12 month+4 month as a grace period) gap between HbA1c measurements because these patients were likely to have received DM care elsewhere; and (4) with IgM MGUS since this subtype of MGUS usually progresses to lymphoma [6, 32]. The follow-up period was defined as the date of MGUS diagnosis to the date of MM diagnosis, death, or censoring (4/15/2025), whichever occurred first.

This study was approved by the Institutional Review Boards at Washington University School of Medicine and the St. Louis VA Medical Center.

### Exposure

The exposure was time-varying poor diabetic control, defined as a binary variable – 1: HbA1c > 7% versus 0: HbA1c ≤ 7% during the follow-up at 1-day intervals. The 7% cutoff was chosen to align with the common glycemic index goal provided by the American Diabetes Association (ADA) for DM [33]. Daily HbA1c values were imputed based on consecutive HbA1c measurements. First, each available HbA1c value was applied retroactively as the value for the 90 days preceding it, or all of the days following the prior measurement if the gap between the two measurements was ≤90 days. The timeframe of 90 days was chosen due to clinical guidelines recommending 3-month follow-up frequency [34]. If the gap between two consecutive measurements was >90 days, the remaining interval (i.e., days outside of the 90-day period) was then imputed based on the concordance of consecutive HbA1c values and categorized into HbA1c > 7% or ≤7%. If consecutive HbA1c values were both >7% or both ≤7%, then the days outside of the 90-day period were categorized as the same controlled diabetes status. If the HbA1c went from >7% to ≤7%, then the first third of the remaining interval were assigned to HbA1c > 7% and the remaining two-thirds of the interval were assigned to HbA1c ≤ 7%; otherwise, the first half of the interval was assigned to HbA1c ≤ 7% and the other half to HbA1c > 7% (see an example illustration in Supplementary Fig. 1). The rationale for this assumption was because the change from HbA1c > 7% to ≤7% is likely due to the medication effect which is immediate versus the change from HbA1c ≤ 7% to >7% likely from lifestyle factors, which gradually increase the HbA1c. For patients without an HbA1c measurement <90 days after MGUS diagnosis and/or on the day of the end of follow-up, we retrieved one HbA1c record before and one after the follow-up period, if available, to ensure complete exposure status for the full follow-up period.

### Outcome

The primary outcome was time (in days) from MGUS diagnosis to progression to MM. MM was confirmed by a published and validated NLP algorithm utilizing diagnostic data and the receipt of myeloma-specific treatment (Supplementary Table 2). The NLP algorithm achieved accuracy/F1 scores for MGUS and MM of 93%/96% and 99%/96%, respectively [31].

### Covariates

The covariates included DM type (T2DM without and with insulin use at time of MGUS, T1DM, body mass index (BMI) category (underweight [BMI < 18.5 kg/m^2^], normal weight [BMI 18.5–25 kg/m^2^], overweight [BMI 25–30 kg/m^2^] and obese [BMI ≥ 30 kg/m^2^]), M-protein level (0, <1.5 g/dL, ≥1.5 g/dL, not quantified), age, DM medication use (sodium-glucose cotransporter-2 inhibitor [SGLT-2i], metformin, GLP-1RA, sulfonylurea [SU], thiazolidinediones [TZD], dipeptidyl peptidase-4 inhibitor [DPP-4i]), Charlson Comorbidity Index (CCI), anemia, and chronic kidney disease (CKD), all collected at the time of MGUS diagnosis ( < 1 year before or after MGUS diagnosis) along with sex (female or male), MGUS subtype (IgG, IgA, light-chain), race/ethnicity (Hispanic, NHB, NHW, Other, Unknown), and MGUS diagnosis year. Anemia and CKD were captured by ICD-9/10 code (Supplementary Table 1). Race/ethnicity information was self-reported as recorded in the electronic health records in the VHA. Objectively measured height and weight were collected from Corporate Data Warehouse domains in the VHA. The weight value closest to MGUS diagnosis and the height value with the highest frequency were utilized to calculate BMI. DM medication use was identified <1 year before or after MGUS diagnosis. The CCI was calculated with comorbidities present (excluding DM) identified by ICD codes <1 year prior to MGUS diagnosis.

### Statistical analyses

The baseline characteristics, stratified by HbA1c > 7% versus ≤7% at MGUS diagnosis, were summarized in percentages for categorical variables and in medians and minimum-maximum ranges for continuous variables. We categorized missing values as “unknown” to preserve the sample size. We conducted χ2 tests to compare percentages for categorical variables and Kruskal-Wallis tests to compare medians for continuous variables between the groups.

The association between time-varying poor diabetic control and the progression to MM was estimated by multivariable Fine-Gray subdistribution hazard model, with death as a competing event and reported as a multivariable-adjusted hazard ratio (aHR) along with the 95% confidence interval (CI) [35]. To ensure the balance of the covariates between the two groups, all aforementioned analyses were adjusted by inverse probability of treatment weighting (IPTW) [36, 37]. The weights were computed via propensity scores of having a HbA1c level >7% at the time of MGUS diagnosis predicted from logistic regression with all of the aforementioned covariates [38]. Covariate balance after the IPTW adjustment was examined by comparing the standardized mean differences (SMD) before and after IPTW [39].

Negative control methods were used to evaluate the impact of potential unmeasured confounding [40]. Marital status was used in the negative control exposure analysis since studies have not shown its association with MGUS progression [8]. Autoimmune disease after MGUS diagnosis was used in the negative control outcome analysis since studies have shown that autoimmune disease and MM share similar potential sources of bias but autoimmune disease cannot plausibly be related to uncontrolled diabetes.

All statistical tests were two-tailed with statistical significance determined by an alpha level of 0.05. All statistical analyses were performed using SAS version 9.2 (SAS Institute Inc., Cary, NC).

### Subgroup analyses

As a prior study demonstrated increased cancer risk with higher insulin dose levels [30], we conducted subgroup analyses stratified by DM type: T1DM and T2DM with and without insulin use during follow-up. T2DM was stratified by insulin use status throughout follow-up because insulin use can be a confounder given that insulin and its synthetic analogs have proliferative effects [23–25, 41, 42], and there is a wide range of total daily insulin doses.

### Sensitivity analyses

Within each of the two T2DM subgroups with and without insulin use during the follow-up period, sensitivity analyses were performed varying the binary HbA1c cutoffs for uncontrolled diabetes to HbA1c > 6.5%, >7.5%, >8%, and >9% to assess for dose-response relationships, because there are a variety of HbA1c targets depending on patients’ health status and comorbidities. The sensitivity analyses were not performed in the T1DM subgroup due to the small sample size.

## Results

A total of 19,189 patients with NLP-confirmed MGUS diagnosis from 10/1/1999-12/31/2023 with a prior DM diagnosis were identified. We excluded patients without body weight measurements (*n* = 547) or any HbA1c measurements between MGUS and the end of follow-up (*n* = 2412), patients with >16-month gap between HbA1c measurements (*n* = 2897), and patients with IgM MGUS (*n* = 2272). This led to a final analytic cohort of 11,061 patients (Fig. 1), among whom 6195 had HbA1c ≤ 7% and 4866 had HbA1c > 7% at MGUS diagnosis.

The median age at MGUS diagnosis in the HbA1c ≤ 7% group was 72.3 (range: 34.4–99.7) years versus 71.2 (range: 36.1–98.4) years in the HbA1c > 7% group (p < 0.0001; Table 1). The HbA1c ≤ 7% group had a lower percentage of male patients compared to the HbA1c > 7% group (96.9% versus 97.6%; *p* = 0.03). Race/ethnicity distribution was not statistically significant different between the two groups (*p* = 0.22). The median follow-up was 37 months (range: 1–288) for the HbA1c ≤ 7% group and 41 months (range: 1–292) for the HbA1c > 7% group (*p* = 0.05). Progression occurred in 1334 patients at a median time of 16 months (range: 1–206) for the HbA1c ≤ 7 group (*n* = 725) and 12 months (range: 1–176) for the HbA1c > 7 group (*n* = 609; *p* = 0.04).

**Table 1:** Baseline characteristics of the analytic cohort of patients in the Veterans Health Administration diagnosed with MGUS with a prior diagnosis of DM, 1999–2023.

	HbA1c^a^ ≤ 7 at MGUS diagnosis	HbA1c^a^ > 7 at MGUS diagnosis	*P*-value
	(*n* = 6195)	(*n* = 4866)	
DM Type^b^			
T2DM on insulin at MGUS diagnosis	1052 (17.0%)	1083 (22.3%)	<0.0001
T2DM not on insulin at MGUS diagnosis	5004 (80.8%)	3655 (75.1%)	
T1DM	139 (2.2%)	128 (2.6%)	
BMI Category^b^			
Unknown	469 (7.6%)	408 (8.4%)	<0.0001
Underweight	48 (0.8%)	27 (0.6%)	
Normal weight	937 (15.1%)	590 (12.1%)	
Overweight	1845 (29.8%)	1308 (26.9%)	
Obese	2896 (46.8%)	2533 (52.1%)	
M-protein^b^			
0^c^	612 (9.9%)	475 (9.8%)	0.43
<1.5 g/dL	3312 (53.5%)	2656 (54.6%)	
>=1.5 g/dL	374 (6.0%)	279 (5.7%)	
Not quantified^d^	1097 (17.7%)	880 (18.1%)	
Unknown	800 (12.9%)	576 (11.8%)	
Sex			
Female	191 (3.1%)	117 (2.4%)	0.03
Male	6004 (96.9%)	4749 (97.6%)	
MGUS Type^b^			
IgA	802 (13.0%)	586 (12.0%)	0.28
IgG	4451 (71.9%)	3556 (73.1%)	
Light-chain	942 (15.2%)	724 (14.9%)	
Race/Ethnicity			
Hispanic	212 (3.4%)	196 (4.0%)	0.22
Non-Hispanic Black	2045 (33.0%)	1570 (32.3%)	
Non-Hispanic White	3392 (54.8%)	2640 (54.3%)	
Other	99 (1.6%)	96 (2.0%)	
Unknown	447 (7.2%)	364 (7.5%)	
SGLT-2i use^b^	452 (7.3%)	739 (15.2%)	<0.0001
Metformin use^b^	2733 (44.1%)	2514 (51.7%)	<0.0001
Insulin use^b^	2842 (45.9%)	3591 (73.8%)	<0.0001
GLP-1RA use^b^	246 (4.0%)	512 (10.5%)	<0.0001
SU use^b^	2037 (32.9%)	2144 (44.1%)	<0.0001
TZD use^b^	281 (4.5%)	367 (7.5%)	<0.0001
DPP-4i use^b^	364 (5.9%)	612 (12.6%)	<0.0001
Anemia	2349 (37.9%)	1614 (33.2%)	<0.0001
CKD	2359 (38.1%)	2106 (43.3%)	<0.0001
Progression			
No progression, No Death	2558 (41.3%)	2004 (41.2%)	0.41
Confirmed progression	725 (11.7%)	609 (12.5%)	
Death without progression	2912 (47.0%)	2253 (46.3%)	
**Median (min-max)**			
MGUS diagnosis age (years)	72.3 (34.4–99.7)	71.2 (36.1–98.4)	<0.0001
HbA1c^a^ (%)	6.3 (4.0–7.0)	7.9 (7.1–16.8)	<0.0001
Charlson Comorbidity Index (excluding T2DM and T1DM)^b^	3 (0–16)	2 (0–16)	0.002
MGUS diagnosis year	2017 (1999–2023)	2017 (1999–2023)	0.08
Follow-up time, month	37 (1–288)	41 (1–292)	0.05
Time to Progression, month	16 (1–206)	12 (1–176)	0.04

*HbA1c* glycated hemoglobin, *DM* diabetes mellitus, *T2DM* type 2 DM, *T1DM* type 1 DM, *BMI* body mass index, *M-protein* monoclonal protein, *MGUS* monoclonal gammopathy of undetermined significance, *IgA* immunoglobulin A, *IgG* immunoglobulin G, *SGLT-2i* sodium glucose cotransporter-2 inhibitor, *GLP-1RA* glucagon-like peptide-1, *SU* sulfonylurea, *TZD* thiazolidinediones, *DPP-4i* dipeptidyl peptidase-4 inhibitor.

^a^The closest value within 1 year before or after MGUS diagnosis.

^b^This value was obtained within 1 year before or after MGUS diagnosis.

^c^This means that the monoclonal protein was reported as “0” on the SPEP report.

^d^This means that the SPEP specified a monoclonal protein but did not quantify the amount.

The proportions of T2DM on insulin at MGUS, T2DM not on insulin, and T1DM were 17.0%, 80.8%, and 2.2% in the HbA1c ≤ 7% group, versus 22.3%, 75.1%, and 2.6% in the HbA1c > 7% group (*p* < 0.0001). The proportions of underweight, normal weight, overweight, obese, and unknown BMI were 0.8%, 15.1%, 29.8%, 46.8%, and 7.6% in the HbA1c ≤ 7% group, versus 0.6%, 12.1%, 26.9%, 52.1%, and 8.4% in the HbA1c > 7% group (*p* < 0.0001). At MGUS, patients in the HbA1c ≤ 7% group had a higher CCI and a higher proportion of anemia; in contrast, patients in the HbA1c > 7% group had a higher prevalence of CKD and more likely to use anti-diabetic medications. The two groups were not statistically significantly different in MGUS diagnosis year, M-protein levels and immunoglobulin subtypes.

In the overall cohort, HbA1c > 7% was associated with an increased risk of progression: aHR: 1.15, 95% CI 1.01–1.32, *p* = 0.04; Table 2). Other statistically significant variables were M-protein ≥1.5 g/dL (aHR 6.22, 95% CI 5.23–7.40) and listed as not quantified (aHR 1.26, 95% CI 1.05–1.52), compared to M-protein <1.5 g/dL; age (aHR 0.98, 95% CI 0.98–0.99 per 1 year increase); IgA MGUS (aHR 1.84, 95% CI 1.56–2.16), compared to IgG; NHB race/ethnicity (aHR 1.41, 95% CI 1.22–1.63), compared to NHW; CCI (aHR 1.05, 95% CI 1.03–1.08 per 1 score increase); CKD (aHR 0.82, 95% CI 0.70–0.97), compared to no CKD; and MGUS diagnosis year (aHR 0.98, 95% CI 0.97–0.99 per 1 year increase). When evaluating covariate balance with the IPTW, all covariates achieved an SMD of <0.2, indicating that the IPTW successfully balanced the covariates between the two groups (Supplementary Fig. 2). Negative control exposure and outcome analyses did not show any statistically significant associations between marital status and the risk of progression of MGUS to MM or between HbA1c status and the risk of developing autoimmune disease (Supplementary Tables 3 and 4).

**Table 2:** Multivariable IPTW-adjusted analysis of the risk of progression to MM in patients diagnosed with MGUS with a prior diagnosis of DM (*n* = 11,061), 1999–2023.

Variable	aHR (95% CI)	*P*-value
Time-varying HbA1c		
≤7%	1.00 (ref.)	
>7%	1.15 (1.01–1.32)	0.04
DM Type^a^		
T2DM without insulin	1.00 (ref.)	
T2DM on insulin	1.05 (0.84–1.30)	0.68
T1DM	0.74 (0.47–1.17)	0.2
BMI Category^a^		
Underweight	0.69 (0.31–1.54)	0.37
Normal Weight	1.00 (ref.)	
Overweight	0.99 (0.80–1.22)	0.89
Obese	0.94 (0.76–1.15)	0.54
M-Protein Level^a^		
Not quantified	1.26 (1.05–1.52)	0.01
0	0.79 (0.59–1.07)	0.13
>0 and <1.5 g/dL	1.00 (ref.)	
≥1.5 g/dL	6.22 (5.23–7.40)	<0.0001
Sex		
Female	1.00 (ref.)	
Male	1.32 (0.87–2.01)	0.2
MGUS Type		
IgG	1.00 (ref.)	
IgA	1.84 (1.56–2.16)	<0.0001
Light-chain	0.92 (0.73–1.16)	0.49
Race/Ethnicity		
Non-Hispanic White	1.00 (ref.)	
Non-Hispanic Black	1.41 (1.22–1.63)	<0.0001
Hispanic	1.15 (0.80–1.66)	0.45
Other	0.66 (0.35–1.24)	0.2
Unknown	1.18 (0.91–1.52)	0.21
Age^a^	0.98 (0.98–0.99)	<0.0001
SGLT-2i use^a^	1.02 (0.77–1.35)	0.87
Metformin use^a^	1.07 (0.91–1.26)	0.41
GLP-1RA use^a^	0.95 (0.68–1.32)	0.75
SU use^a^	0.96 (0.82–1.12)	0.58
TZD use^a^	0.74 (0.55–1.00)	<0.05
DPP-4i use^a^	1.21 (0.94–1.54)	0.13
CCI (excluding T2DM and T1DM)^a^	1.05 (1.03–1.08)	0.0002
Anemia	1.11 (0.96–1.28)	0.16
CKD	0.82 (0.70–0.97)	0.02
MGUS diagnosis year	0.98 (0.97–0.99)	0.002

*HbA1c* glycated hemoglobin, *DM* diabetes mellitus, *T2DM* type 2 DM, *T1DM* type 1 DM, *BMI* body mass index, *M-protein* monoclonal protein, *MGUS* monoclonal gammopathy of undetermined significance, *IgA* immunoglobulin A, *IgG* immunoglobulin G, *SGLT-2i* sodium glucose cotransporter-2 inhibitor, *GLP-1RA* glucagon-like peptide-1 agonist, *SU* sulfonylurea, *TZD* thiazolidinediones, *DPP-4i* dipeptidyl peptidase-4 inhibitor, *CCI* Charlson Comorbidity Index, *CKD* chronic kidney disease.

^a^This value was obtained within 1 year before or after MGUS diagnosis.

In the subgroup of T2DM patients with insulin use during follow-up (*n* = 7070), HbA1c > 7% was also associated with increased risk: aHR 1.46 (95% CI 1.19–1.78; Table 3). In the subgroup of T2DM patients without insulin use during the follow-up period (*n* = 3724), HbA1c > 7% was associated with increased risk: aHR 1.42 (95% CI 1.14–1.77). In the T1DM cohort (*n* = 267), HbA1c > 7% was not associated with increased risk: aHR 1.54 (95% CI 0.55–4.31).

**Table 3:** Multivariable IPTW-adjusted analysis of the risk of progression to MM in the Veterans Health Administration in MGUS patients with Type II Diabetes Mellitus with or without insulin use during the follow-up period or Type I Diabetes Mellitus, 1999–2023.

	T2DM without insulin use during follow-up (*n* = 3 724)		T2DM with insulin use during follow-up (*n* = 7 070)		T1DM (*n* = 267)	
Variable	aHR (95% CI)	*P*-value	aHR (95% CI)	*P*-value	aHR (95% CI)	*P*-value
Time-varying HbA1c						
<=7%	1.00 (ref.)		1.00 (ref.)		1.00 (ref.)	
>7%	1.42 (1.14–1.77)	0.002	1.46 (1.19–1.78)	0.0002	1.54 (0.55–4.31)	0.41
BMI Category^a^						
Underweight	0.46 (0.08–2.67)	0.39	0.83 (0.32–2.16)	0.7	NE	NE
Normal Weight	1.00 (ref.)		1.00 (ref.)		1.00 (ref.)	
Overweight	0.95 (0.69–1.29)	0.73	1.07 (0.79–1.45)	0.64	0.14 (0.03–0.70)	0.02
Obese	1.06 (0.79–1.43)	0.7	0.91 (0.68–1.22)	0.52	0.36 (0.09–1.44)	0.15
M-Protein Level^a^						
Not quantified	1.41 (1.07–1.85)	0.01	1.23 (0.95–1.58)	0.12	3.88 (0.55–27.60)	0.18
0	0.67 (0.42–1.06)	0.09	0.88 (0.59–1.32)	0.53	2.27 (0.13–41.05)	0.58
>0 and <1.5 g/dL	1.00 (ref.)		1.00 (ref.)		1.00 (ref.)	
≥1.5 g/dL	6.00 (4.65–7.73)	<0.0001	6.01 (4.69–7.69)	<0.0001	41.59 (6.55–263.86)	<0.0001
Sex						
Female	1.00 (ref.)		1.00 (ref.)		1.00 (ref.)	
Male	1.49 (0.84–2.64)	0.17	1.36 (0.69–2.67)	0.37	0.16 (0.01–3.56)	0.25
MGUS Type^a^						
IgG	1.00 (ref.)		1.00 (ref.)		1.00 (ref.)	
IgA	2.45 (1.96–3.07)	<0.0001	1.51 (1.18–1.92)	0.0009	1.93 (0.52–7.19)	0.33
Light–chain	0.96 (0.68–1.36)	0.82	0.87 (0.63–1.21)	0.4	0.20 (0.00–10.33)	0.43
Race/Ethnicity						
Non–Hispanic White	1.00 (ref.)		1.00 (ref.)		1.00 (ref.)	
Non–Hispanic Black	1.71 (1.38–2.11)	<0.0001	1.27 (1.03–1.56)	0.03	3.22 (1.08–9.61)	0.04
Hispanic	1.18 (0.68–2.03)	0.56	1.12 (0.67–1.88)	0.67	NE	NE
Other	0.35 (0.09–1.36)	0.13	0.97 (0.47–2.01)	0.94	NE	NE
Unknown	0.69 (0.45–1.07)	0.1	1.40 (0.99–1.97)	0.06	0.74 (0.05–10.70)	0.83
Age^a^	0.97 (0.96–0.98)	<0.0001	0.98 (0.97–0.99)	0.0005	0.98 (0.93–1.03)	0.37
SGLT–2i use^a^	0.88 (0.56–1.37)	0.56	1.15 (0.79–1.68)	0.47	NE	NE
Metformin use^a^	0.91 (0.74–1.12)	0.37	1.14 (0.93–1.40)	0.21	NE	NE
GLP–1RA use^a^	1.87 (1.14–3.07)	0.01	0.70 (0.43–1.14)	0.15	NE	NE
SU use^a^	1.12 (0.91–1.38)	0.28	0.74 (0.61–0.91)	0.004	NE	NE
TZD use^a^	0.71 (0.42–1.22)	0.22	0.87 (0.59–1.26)	0.45	NE	NE
DPP–4i use^a^	1.21 (0.83–1.77)	0.32	1.19 (0.85–1.66)	0.32	NE	NE
CCI	1.10 (1.06–1.14)	<0.0001	1.07 (1.03–1.11)	0.0002	0.86 (0.68–1.09)	0.21
(excluding T2DM and T1DM)^a^						
Anemia	1.16 (0.93–1.44)	0.19	1.12 (0.92–1.36)	0.26	3.03 (0.86–10.72)	0.08
CKD	0.82 (0.62–1.07)	0.15	0.93 (0.75–1.16)	0.53	1.78 (0.55–5.79)	0.34
MGUS diagnosis year	0.95 (0.93–0.97)	<0.0001	0.98 (0.96–1.00)	0.03	0.98 (0.90–1.07)	0.64

For the T1DM subgroup, certain covariates were too infrequent to calculate and were listed as not evaluable (NE).

*HbA1c* glycated hemoglobin, *DM* diabetes mellitus, *T2DM* type 2 DM, *T1DM* type 1 DM, *BMI* body mass index, *NE* not evaluable, *M-protein* monoclonal protein, *MGUS* monoclonal gammopathy of undetermined significance, *IgA* immunoglobulin A, *IgG* immunoglobulin G, *SGLT-2i* sodium glucose cotransporter-2 inhibitor, *GLP-1RA* glucagon-like peptide-1 agonist, *SU* sulfonylurea, *TZD* thiazolidinediones, *DPP-4i* dipeptidyl peptidase-4 inhibitor, *CCI* Charlson Comorbidity Index, *CKD* chronic kidney disease.

^a^This value was obtained within 1 year before or after MGUS diagnosis.

Sensitivity analysis varying the HbA1c set points to >6.5, >7.5%, >8%, and >9% within the subgroup of T2DM without insulin use throughout the follow-up period demonstrated larger effect size by set points: HbA1c > 6.5: aHR 1.24 (95% CI 1.00–1.53, *p* < 0.05); >7.5: aHR 1.52 (95% CI 1.16–1.99); >8: 1.74 (95% CI 1.27–2.39); >9: aHR 2.07 (95% CI 1.30–3.28), respectively. The same analysis performed in T2DM with insulin use during follow-up did not demonstrate an increase in effect sizes (Supplementary Table 5).

## Discussion

To our knowledge, this is the first study to explore the relationship between poor diabetic control, defined as Hba1c > 7% and progression of MGUS to MM. In our overall cohort, poorly controlled DM was associated with a 15% increased progression risk. In T2DM with and without insulin use during the follow-up: poorly controlled DM was associated with 46% and 42% increased risks of progression, respectively.

We also found that in the subgroup of T2DM without insulin use during follow-up, using higher HbA1c set points (6.5–9%) resulting in higher associations between poorly controlled diabetes and progression risk suggesting a dose-response relationship [30]. This could be related to high levels of endogenous insulin promoting myelomagenesis [21–26] and the elevated glucose contributing to the avoidance of cell death and the development of cancer [43].

Our study contributes to the literature by defining the progression risk associated with the level of diabetic control, instead of relying only on the clinical diagnosis of DM [13–18, 20], since complications of DM are generally associated with the degree of diabetic control over time. Current guidelines for the management of MGUS do not address the frequency of glycemic index monitoring when patients have concurrent DM and MGUS [44]. This study suggests that for patients with both DM and MGUS, regular monitoring of diabetes with routinely collected HbA1c levels could inform progression risk and potentially identify an opportunity for intervention through optimization of DM control.

Interestingly, deviating from prior studies [8, 45–48], there is no evidence demonstrating that high BMI at MGUS diagnosis were associated with progression risk in this cohort with MGUS and a prior diagnosis of T2DM. We postulate that obesity is highly correlated with the incidence of both T2DM and MM [10, 27]. Therefore, as noted in a prior study, the increased risk of progression to MM associated with obesity in patients with both T2DM and MGUS is not as prominent as those with MGUS regardless of T2DM status [27].

The strengths of this study include the quantification of the association between poorly controlled DM and MGUS progression, contributing to the link between DM and MM [13–18, 20]. Another strength is the use of population-level data from a national database with several key advantages, including a centralized national electronic health record system that provides comprehensive laboratory and diagnostic information. Additionally, we used data linked to the Department of Defense, Centers for Medicare & Medicaid Services (CMS), and Purchased Care to capture all diagnoses and medications as well as a broad spectrum of healthcare services within and outside of the VHA network. We also utilized a published and validated NLP-assisted machine learning-based models to verify MGUS and MM diagnoses [31].

The limitations include the extrapolation of HbA1c values for each day from the measured serum HbA1c laboratory testing values before/after and during the follow-up period. However, as an observation study HbA1c was not measured every day, we had to develop an algorithm to proxy for the daily values via discussions with VHA clinicians (MWS, TST). Moreover, as this is a population-based study, it would be unable to identify molecular and biological pathways driving the association between poorly controlled diabetes and progression to MM. Regardless, many prior studies have assessed the mechanisms for MGUS progression [22, 23, 25, 49]. Furthermore, given the retrospective nature of this study, we acknowledge that residual confounding from unmeasured factors, e.g., medication adherence, healthcare access, socioeconomic status (SES), still exist leading to potential biases that could possibly arising from differential monitoring between groups modifying diagnoses time of MGUS and/or progression [20]. For this, we examined median outpatient visits in the year prior to MGUS diagnosis for HbA1c > 7% versus ≤7% (26.0 [interquartile range (IQR) 12.0–42.0] versus 25.0 [IQR 15.0–42.0], *p* = 0.58) and found no evidence on differences between the two groups. While good glycemic control may indicate more frequent care, the converse may also be true as the ADA recommends more frequent monitoring for poorly controlled DM. Additionally, studies have not found SES to be associated with MGUS progression risk in the VHA population [8], possibly due to low barriers to care, making this population ideal for studying progression of MGUS to MM. For the concern about residual confounding due to the retrospective nature of this study, we also performed negative control analyses to reassure a minimized concern. Finally, since the VHA population is not the US general population, our results may not be generalizable to other populations [50–52].

## Conclusion

For patients with DM and MGUS, poor diabetic control is associated with a higher MGUS to MM progression risk. This suggests that patients with MGUS and DM may benefit from HbA1c monitoring and diabetes management at regular intervals in addition to other laboratory assessments for their MGUS. Future studies should prospectively evaluate whether improved diabetic control in patients with MGUS may reduce progression risk.

## Supplementary information

Supplementary Files

## Data Availability

The data obtained from the VHA for the analyses in this study contain patient health information, including Social Security numbers and addresses. The institutional review board (IRB)–approved study protocol allows us to share analysis results only in an aggregate format. We do not have IRB approval to share deidentified data. We also have the following policies in place that do not allow us to share the data: In our original IRB application under “Data Management and Access Plan”: Will final data sets underlying the publications be shared outside the US Department of Veterans Affairs (VA)? No. The final data sets contain patient health information and will not be shared outside of the VA. In our data use agreement with VA Information Resource Center for use of VA and Centers for Medicare & Medicaid Services (CMS) data: Data security: VA/CMS data will be protected in accordance with the project’s VA/CMS Data Security Compliance Form and may be stored and used only on VA Office of Information and Technology–managed network servers located at: VA facility: VA Austin Information Technology Center, Austin, Texas. VA Informatics and Computing Infrastructure workspace: ORD_Chang_202011003D. In our data access permissions: Access to these data will be restricted to only VA-approved project employees whose roles and responsibilities necessitate access to these data (hereinafter referred to as authorized data users) and who have signed and submitted a VA/CMS Rules of Behavior Agreement.
