# Supplementary material for: Association between diabetic control and progression of monoclonal gammopathy of undetermined significance to multiple myeloma
Source: Blood Cancer J. 2026 Aug 3;16(1):135. doi: 10.1038/s41408-026-01592-x (PMC13462740; doi:10.1038/s41408-026-01592-x)
Supplement: Supplementary file 1 [file 41408_2026_1592_MOESM1_ESM.docx]

**SUPPLEMENTAL**

**Supplemental Table 1.** List of Diabetes Mellitus ICD-9/10 Codes.

| Type I Diabetes Mellitus | | Type II Diabetes Mellitus | |
| --- | --- | --- | --- |
| ICD-9 Codes | ICD-10 Codes | ICD-9 Codes | ICD-10 Codes |
| 250.01, 250.03, 250.11, 250.13, 250.21, 250.23, 250.31, 250.33, 250.41, 250.43, 250.51, 250.53, 250.61, 250.63, 250.71, 250.73, 250.81, 250.83, 250.91, 250.93 | E10.10,  E10.11,  E10.21,  E10.22,  E10.29,  E10.311,  E10.319,  E10.321,  E10.3211,  E10.3212,  E10.3213,  E10.3219,  E10.329,  E10.3291,  E10.3292,  E10.3293,  E10.3299,  E10.331,  E10.3311,  E10.3312,  E10.3313,  E10.3319,  E10.339,  E10.3391,  E10.3392,  E10.3393,  E10.3399,  E10.341,  E10.3411,  E10.3412,  E10.3413,  E10.3419,  E10.349,  E10.3491,  E10.3492,  E10.3493,  E10.3499,  E10.351,  E10.3511,  E10.3512,  E10.3513,  E10.3519,  E10.3521,  E10.3522,  E10.3523,  E10.3529,  E10.3531,  E10.3532,  E10.3533,  E10.3539,  E10.3541,  E10.3542,  E10.3543,  E10.3549,  E10.3551,  E10.3552,  E10.3553,  E10.3559,  E10.359,  E10.3591,  E10.3592,  E10.3593,  E10.3599,  E10.36,  E10.37X1,  E10.37X2,  E10.37X3,  E10.37X9,  E10.39,  E10.40,  E10.41,  E10.42,  E10.43,  E10.44,  E10.49,  E10.51,  E10.52,  E10.59,  E10.610,  E10.618,  E10.620,  E10.621,  E10.622,  E10.628,  E10.630,  E10.638,  E10.641,  E10.649,  E10.65,  E10.69,  E10.8,  E10.9 | 250.00, 250.02, 250.10, 250.12, 250.20, 250.22, 250.30, 250.32, 250.40, 250.42, 250.50, 250.52, 250.60, 250.62, 250.70, 250.72, 250.80, 250.82, 250.90, 250.92 | E11.00,  E11.01,  E11.10,  E11.11,  E11.21,  E11.22,  E11.29,  E11.311,  E11.319,  E11.321,  E11.3211,  E11.3212,  E11.3213,  E11.3219,  E11.329,  E11.3291,  E11.3292,  E11.3293,  E11.3299,  E11.331,  E11.3311,  E11.3312,  E11.3313,  E11.3319,  E11.339,  E11.3391,  E11.3392,  E11.3393,  E11.3399,  E11.341,  E11.3411,  E11.3412,  E11.3413,  E11.3419,  E11.349,  E11.3491,  E11.3492,  E11.3493,  E11.3499,  E11.351,  E11.3511,  E11.3512,  E11.3513,  E11.3519,  E11.3521,  E11.3522,  E11.3523,  E11.3529,  E11.3531,  E11.3532,  E11.3533,  E11.3539,  E11.3541,  E11.3542,  E11.3543,  E11.3549,  E11.3551,  E11.3552,  E11.3553,  E11.3559,  E11.359,  E11.3591,  E11.3592,  E11.3593,  E11.3599,  E11.36,  E11.37X1,  E11.37X2,  E11.37X3,  E11.37X9,  E11.39,  E11.40,  E11.41,  E11.42,  E11.43,  E11.44,  E11.49,  E11.51,  E11.52,  E11.59,  E11.610,  E11.618,  E11.620,  E11.621,  E11.622,  E11.628,  E11.630,  E11.638,  E11.641,  E11.649,  E11.65,  E11.69,  E11.8,  E11.9 |

**Supplemental Table 2.** List of myeloma-specific treatment terms.

abecma

belamaf

belantamab

belmaf

bendamustine

blenrep

bortezomib

carfilzomib

carvykti

cevostamab

ciltacabtagene

cilta-cel

cyclophosphamide

cytoxan

daratumumab

darzalex

elotuzumab

elranatamab

empliciti

evomela

farydak

idecabtagene

ide-cel

isatuximab

ixazomib

kyprolis

lenalidomide

melflufen

melphalan

ninlaro

panobinostat

pomalidomide

pomalyst

revlimid

sarclisa

selinexor

talquetamab

teclistamab

tecvayli

thalidomide

thalomid

velcade

venclexta

venetoclax

xpovio

**Supplemental Table 3.** Multivariable negative control exposure analysis of marital status and the risk of developing MM in the Veterans Health Administration in the overall cohort and with the subgroups of Type II Diabetes Mellitus with or without insulin use during the follow-up period

| **Variable** | **Overall (N=11,061)** | | **T2DM without insulin use during follow-up (N=3 724)** | | **T2DM with insulin use during follow-up (N=7 070)** | | |
| --- | --- | --- | --- | --- | --- | --- | --- |
|  | **aHR (95% CI)** | **P-value** | **aHR (95% CI)** | **P-value** | | **aHR (95% CI)** | **P-value** |
| **Marital Status** | |  |  |  | |  |  |
| Married | 1.00 (ref.) |  | 1.00 (ref.) |  | | 1.00 (ref.) |  |
| Single or never married | 0.93 (0.76-1.14) | 0.49 | 0.94 (0.67-1.30) | 0.69 | | 0.94 (0.72-1.23) | 0.64 |
| Divorced, separated, or widowed | 0.91 (0.81-1.03) | 0.14 | 0.95 (0.79-1.15) | 0.63 | | 0.90 (0.77-1.05) | 0.16 |
| Unknown | 0.63 (0.30-1.36) | 0.24 | 1.37 (0.57-3.28) | 0.48 | | 0.36 (0.11-1.20) | 0.09 |
| The exposure, marital status (married, single or never married, divorced/separated/widowed, or unknown, were examined by multivariable-adjusted hazard ratio [aHR] using a stratified Fine-Gray subdistribution hazard model, with death as a competing event. The covariates included DM type (T2DM without insulin use, T2DM with insulin use, and T1DM), BMI category (underweight, normal weight, overweight, obese), M-protein level (<1.5 g/dL or ≥1.5g/dL), MGUS diagnosis age, sex (female or male), MGUS subtype (IgG, IgA, or light-chain), race/ethnicity (Hispanic, Non-Hispanic Black, Non-Hispanic White, Other), DM medication usage (sodium-glucose cotransporter-2 inhibitor [SGLT-2i], metformin, glucagon-like peptide-1 agonist [GLP-1RA], sulfonylurea [SU], thiazolidinediones [TZD], dipeptidyl peptidase-4 inhibitor [DPP-4i]), Charlson Comorbidity Index (CCI), anemia, and chronic kidney disease (CKD), and MGUS diagnosis year, all collected at the time of MGUS diagnosis.  Abbreviations: diabetes mellitus, DM; type 2 DM, T2DM; type 1 DM, T1DM; body mass index, BMI; monoclonal protein, M-protein; monoclonal gammopathy of undetermined significance, MGUS; immunoglobulin A, IgA; immunoglobulin G, IgG; sodium glucose cotransporter-2 inhibitor, SGLT-2i; glucagon-like peptide-1 agonist, GLP-1RA; sulfonylurea, SU; thiazolidinediones, TZD; dipeptidyl peptidase-4 inhibitor, DPP-4i; Charlson Comorbidity Index, CCI; chronic kidney disease, CKD. | | | | | | | |

**Supplemental Table 4.** Multivariable negative control outcome analysis of the risk of developing autoimmune disease in the Veterans Health Administration in the overall cohort and with the subgroups of Type II Diabetes Mellitus with or without insulin use during the follow-up period.

| **Variable** | **Overall (N=10 992)** | | **T2DM without insulin use during follow-up (N=3 701)** | | **T2DM with insulin use during follow-up (N=7 030)** | | | |
| --- | --- | --- | --- | --- | --- | --- | --- | --- |
|  | **aHR (95% CI)** | **P-value** | **aHR (95% CI)** | **P-value** | | **aHR (95% CI)** | **P-value** | |
| **HbA1c** | |  |  |  | |  |  | |
| ≤7% | 1.00 (ref.) |  | 1.00 (ref.) |  | | 1.00 (ref.) |  | |
| >7% | 0.73 (0.49-1.11) | 0.14 | 0.44 (0.12-1.57) | 0.21 | | 0.77 (0.47-1.26) | 0.3 | |
| The exposure, time-varying HbA1c>7%, was examined by multivariable-adjusted hazard ratio [aHR] using a stratified Fine-Gray subdistribution hazard model, with death as a competing event. The covariates included DM type (T2DM without insulin use, T2DM with insulin use, and T1DM), BMI category (underweight, normal weight, overweight, obese), M-protein level (<1.5 g/dL or ≥1.5g/dL), MGUS diagnosis age, sex (female or male), MGUS subtype (IgG, IgA, or light-chain), race/ethnicity (Hispanic, Non-Hispanic Black, Non-Hispanic White, Other), DM medication usage (sodium-glucose cotransporter-2 inhibitor [SGLT-2i], metformin, glucagon-like peptide-1 agonist [GLP-1RA], sulfonylurea [SU], thiazolidinediones [TZD], dipeptidyl peptidase-4 inhibitor [DPP-4i]), Charlson Comorbidity Index (CCI), anemia, and chronic kidney disease (CKD), and MGUS diagnosis year, all collected at the time of MGUS diagnosis.  The negative control outcome analysis used the development of any autoimmune disease after MGUS diagnosis. The development of autoimmune disease was a composite outcome including Systemic Lupus Erythematosus, Polymyositis, Dermatomyositis, Systemic Sclerosis, Autoimmune Hemolytic Anemia, Pernicious Anemia, Ankylosing Spondylitis, and Rheumatoid Arthritis.  Abbreviations: glycated hemoglobin, HbA1c; diabetes mellitus, DM; type 2 DM, T2DM; type 1 DM, T1DM; body mass index, BMI; monoclonal protein, M-protein; monoclonal gammopathy of undetermined significance, MGUS; immunoglobulin A, IgA; immunoglobulin G, IgG; sodium glucose cotransporter-2 inhibitor, SGLT-2i; glucagon-like peptide-1 agonist, GLP-1RA; sulfonylurea, SU; thiazolidinediones, TZD; dipeptidyl peptidase-4 inhibitor, DPP-4i; Charlson Comorbidity Index, CCI; chronic kidney disease, CKD. | | | | | | | |  |

**Supplemental Table 5.** Sensitivity analysis of a variety of HbA1c cutoffs in patients with Type 2 Diabetes Mellitus and MGUS in the Veterans Health Administration, 1999-2023.

|  | T2DM without insulin during the follow-up | | T2DM with insulin during the follow-up | |
| --- | --- | --- | --- | --- |
| HbA1C | aHR (95% CI) | P-value | aHR (95% CI) | P-value |
| **Continuous** | 1.12 (1.02-1.21) | 0.02 | 1.06 (1.00-1.12) | 0.0482 |
| **> 6.5** | 1.24 (1.00-1.53) | 0.0477 | 1.53 (1.22-1.93) | 0.0003 |
| **> 7** | 1.42 (1.14-1.77) | 0.002 | 1.46 (1.19-1.78) | 0.0002 |
| **> 7.5** | 1.52 (1.16-1.99) | 0.002 | 1.16 (0.95-1.41) | 0.15 |
| **> 8** | 1.74 (1.27-2.39) | 0.0006 | 1.04 (0.83-1.31) | 0.72 |
| **> 9** | 2.07 (1.30-3.28) | 0.002 | 0.78 (0.56-1.10) | 0.15 |

Insulin use or non-use was determined by the presence of any insulin therapy during the follow up period. The increasing HbA1c cutoffs were analyzed by inverse probability of treatment weighted, multivariable-adjusted hazard ratio [aHR] using a stratified Fine-Gray subdistribution hazard model, with death as a competing event. The covariates included BMI category (underweight, normal weight, overweight, obese), M-protein level (<1.5 g/dL or ≥1.5g/dL), MGUS diagnosis age, sex (female or male), MGUS subtype (IgG, IgA, or light-chain), race/ethnicity (Hispanic, Non-Hispanic Black, Non-Hispanic White, Other), DM medication usage (sodium-glucose cotransporter-2 inhibitor [SGLT-2i], metformin, glucagon-like peptide-1 agonist [GLP-1RA], sulfonylurea [SU], thiazolidinediones [TZD], dipeptidyl peptidase-4 inhibitor [DPP-4i]), Charlson Comorbidity Index (CCI), anemia, and chronic kidney disease (CKD), and MGUS diagnosis year, all collected at the time of MGUS diagnosis.

Abbreviations: glycated hemoglobin, HbA1c; adjusted hazard ratio, aHR; confidence interval, CI.

**Supplemental Figure 1** Example illustration of the time-varying HbA1c assignment algorithm

**Time-varying HbA1c Assignment Algorithm**

Daily HbA1c values were imputed based on consecutive HbA1c measurements. First, each available HbA1c value was applied retroactively as the value for the 90 days preceding it or all of the days following the prior measurement if the gap between the two measurements was ≤90 days. The timeframe of 90 days was chosen due to clinical guidelines recommending 3-month follow-up frequency. If the gap between two consecutive measurements was >90 days, the remaining interval (i.e., days outside of the 90-day period) was then imputed based on the concordance of consecutive HbA1c values and categorized into HbA1c >7% or ≤7%. If consecutive HbA1c values were both >7% or both ≤7%, then the days outside of the 90-day period were categorized as the same controlled diabetes status. If the HbA1c went from >7% to ≤7%, then the first third of the remaining interval were assigned to HbA1c >7% and the remaining two-thirds of the interval were assigned to HbA1c ≤7%; otherwise, the first half of the interval was assigned to HbA1c ≤7% and the other half to HbA1c >7% (see an example illustration in Supplemental Figure 1). The rationale for this assumption was because the change from HbA1c >7% to ≤7% is likely due to the medication effect which is immediate versus the change from HbA1c ≤7% to >7% likely from lifestyle factors, which gradually increase the HbA1c. For patients without an HbA1c measurement <90 days after MGUS diagnosis and/or on the day of the end of follow-up, we retrieved one HbA1c record before and one after the follow-up period, if available, to ensure complete exposure status for the full follow-up period.

Abbreviations: glycated hemoglobin, HbA1c; monoclonal gammopathy of undetermined significance, MGUS; multiple myeloma, MM.

**Supplemental Figure 2.** Covariate balance diagnostics

We calculated the standardized mean differences (SMD) to evaluate covariate balance before and after IPTW. All covariates achieved an SMD of <0.2 after weighting, indicating that the IPTW method successfully balanced the two groups (HbA1c >7 vs. ≤7 at MGUS diagnosis).

Overall:

**Standardized mean difference**

| **Covariate** | **Crude SMD** | **Weighted SMD** |
| --- | --- | --- |
| Anemia^†^ | 0.23 | -0.02 |
| BMI^†^ | 0.19 | <0.01 |
| CKD^†^ | 0.23 | -0.02 |
| CCI^†^ | -0.06 | <0.01 |
| DM Type | 0.15 | 0.07 |
| DPP-4i use^†^ | 0.23 | -0.02 |
| Sex | <0.01 | <0.01 |
| GLP-1RA use^†^ | 0.25 | -0.02 |
| Metformin use^†^ | 0.15 | 0.01 |
| Age^†^ | -0.10 | <0.01 |
| MGUS Type | 0.03 | <0.01 |
| MGUS diagnosis year | 0.03 | -0.02 |
| M-protein level | 0.05 | <0.01 |
| Race/Ethnicity | 0.07 | <0.01 |
| SGLT-2i use^†^ | 0.25 | -0.02 |
| SU use^†^ | 0.23 | <0.01 |
| TZD use^†^ | 0.13 | <0.01 |

†This value was obtained within 1 year before or after MGUS diagnosis.
Abbreviations: standardized mean differences, SMD; diabetes mellitus, DM; type 2 DM, T2DM; body mass index, BMI; monoclonal protein, M-protein; monoclonal gammopathy of undetermined significance, MGUS; immunoglobulin A, IgA; immunoglobulin G, IgG; sodium glucose cotransporter-2 inhibitor, SGLT-2i; glucagon-like peptide-1 agonist, GLP-1RA; sulfonylurea, SU; thiazolidinediones, TZD; dipeptidyl peptidase-4 inhibitor, DPP-4i; Charlson Comorbidity Index, CCI; chronic kidney disease, CKD.

T2DM without insulin use during follow-up:

**Standardized mean difference**

| **Covariate** | **Crude SMD** | **Weighted SMD** |
| --- | --- | --- |
| Anemia^†^ | 0.40 | -0.02 |
| BMI^†^ | 0.12 | 0.15 |
| CKD^†^ | 0.40 | -0.02 |
| CCI^†^ | -0.21 | 0.01 |
| DPP-4i use^†^ | 0.40 | -0.02 |
| Sex | -0.05 | <0.01 |
| GLP-1RA use^†^ | 0.26 | -0.02 |
| Metformin use^†^ | 0.32 | <0.01 |
| Age^†^ | -0.05 | 0.03 |
| MGUS Type | 0.11 | 0.03 |
| MGUS diagnosis year | 0.06 | -0.03 |
| M-protein level | 0.14 | 0.05 |
| Race/Ethnicity | 0.17 | 0.02 |
| SGLT-2i use^†^ | 0.35 | -0.01 |
| SU use^†^ | 0.56 | <0.01 |
| TZD use^†^ | 0.16 | <0.01 |

†This value was obtained within 1 year before or after MGUS diagnosis.
Abbreviations: standardized mean differences, SMD; type 2 DM, T2DM; body mass index, BMI; monoclonal protein, M-protein; monoclonal gammopathy of undetermined significance, MGUS; immunoglobulin A, IgA; immunoglobulin G, IgG; sodium glucose cotransporter-2 inhibitor, SGLT-2i; glucagon-like peptide-1 agonist, GLP-1RA; sulfonylurea, SU; thiazolidinediones, TZD; dipeptidyl peptidase-4 inhibitor, DPP-4i; Charlson Comorbidity Index, CCI; chronic kidney disease, CKD.

T2DM with insulin use during follow-up:

**Standardized mean difference**

| **Covariate** | **Crude SMD** | **Weighted SMD** |
| --- | --- | --- |
| Anemia^†^ | 0.18 | -0.01 |
| BMI^†^ | 0.11 | 0.14 |
| CKD^†^ | 0.18 | -0.01 |
| CCI^†^ | -0.15 | <0.01 |
| DPP-4i use^†^ | 0.18 | -0.01 |
| Sex | 0.01 | <0.01 |
| GLP-1RA use^†^ | 0.22 | -0.01 |
| Metformin use^†^ | 0.17 | <0.01 |
| Age^†^ | -0.06 | <0.01 |
| MGUS Type | 0.06 | <0.01 |
| MGUS diagnosis year | 0.18 | -0.01 |
| M-protein level | 0.05 | <0.01 |
| Race/Ethnicity | 0.02 | <0.01 |
| SGLT-2i use^†^ | 0.24 | -0.01 |
| SU use^†^ | 0.10 | <0.01 |
| TZD use^†^ | 0.08 | <0.01 |

†This value was obtained within 1 year before or after MGUS diagnosis.
Abbreviations: standardized mean differences, SMD; type 2 DM, T2DM; body mass index, BMI; monoclonal protein, M-protein; monoclonal gammopathy of undetermined significance, MGUS; immunoglobulin A, IgA; immunoglobulin G, IgG; sodium glucose cotransporter-2 inhibitor, SGLT-2i; glucagon-like peptide-1 agonist, GLP-1RA; sulfonylurea, SU; thiazolidinediones, TZD; dipeptidyl peptidase-4 inhibitor, DPP-4i; Charlson Comorbidity Index, CCI; chronic kidney disease, CKD.
